# The Milk Thistle Seed Cakes and Hempseed Cakes are Potential Feed for Poultry

**DOI:** 10.3390/ani10081384

**Published:** 2020-08-10

**Authors:** Ondrej Stastnik, Leos Pavlata, Eva Mrkvicova

**Affiliations:** Department of Animal Nutrition and Forage Production, Faculty of AgriSciences, Mendel University in Brno, Zemědělská 1, CZ-613 00 Brno, Czech Republic; leos.pavlata@mendelu.cz (L.P.); eva.mrkvicova@mendelu.cz (E.M.)

**Keywords:** *Silybum marianum*, *Cannabis sativa*, pomace, expellers, hens, broilers, poultry nutrition

## Abstract

**Simple Summary:**

In accordance with Commission Regulation (EC) No 726/2004, the use of antibiotics as stimulators of animal growth and performance has been banned in all European Union countries since 2006 (due to the elimination of antibiotic residues from the human food chain). Due to this ban, many scientists are studying alternative approaches to the use of various biologically active substances with a growth-promoting effect. A promising direction is the use of alternative feeds containing bioactive compounds or mixtures of natural origin, or the use of phytoadditives or plant extracts, probiotics, prebiotics, symbiotics or oilseed by-products, such as hempseed cakes and milk thistle seed cakes, in animal nutrition.

**Abstract:**

The aims of this work were to summarize the nutritional value of the milk thistle seed cakes and hempseed cakes and describe the influence on selected performance parameters, metabolism and animal health from inclusion of these non-traditional feeds into diets. It seems more appropriate to apply the extract of the bioactive substances complex to the livestock diets than addition of expellers or other forms of plants processing. The seed expellers, etc. mostly worsened the chickens’ performance parameters with higher doses in diets, while most of the work using the extract yields had positive results on animal performance.

## 1. Introduction

### 1.1. Silybum marianum (L.)

*Silybum marianum* (L.) is an annual or biennial herb naturally occurring in the regions of the Mediterranean and North Africa [1]. The active substances contained in the purple variety of milk thistle, known as the silymarin complex, have hepatoprotective, detoxifying and antioxidant effects, thanks to which parts of this plant are used for the treatment and prevention of liver and bile diseases (hepatitis, cirrhosis) and hence the protection from toxins and various chemicals substances [2].

The highest proportion of active substances of the silymarin complex is found in the seeds, which contain about 70–80% of the silymarin flavonolignans and about 20–30% of chemically undefined substances mainly of the polyphenol structure [2]. According to Kroll et al. [3] the silymarin complex is composed of at least seven flavonolignans and one flavonoid (taxifolin). Milk thistle seeds also contain betaine, trimethyl glycine and essential fatty acids involved in the hepatoprotective and anti-inflammatory effects of the silymarin complex [4,5].

Milk thistle seeds contain 23% ether extract: Oil obtained is a rich source of tocopherols and fatty acids. Milk thistle oil contains 27–64% linoleic acid (C18:2, n-6), 21–50% is oleic acid (C18:1, n-9), 7–14% palmitic acid (C16: 0) and 2–6% stearic acid (C18:0), and α-linolenic acid (C18:3, n-3) is contained in milk thistle seed oil in an amount of 5% [6].

### 1.2. Cannabis sativa

Hemp (*Cannabis sativa* L.) is an annual, up to 2 m tall plant with palm split leaves and multi-sex flowers [7]. Generally, fruits (hempseeds) contain about 25% crude protein, 30% ether extract. The gross energy content of cannabis seeds is 22 MJ/kg [8]. Unhulled hemp seeds contain 25–34% fat and dehulled seeds contain 42–47% fat. Hemp oil, obtained after the pressing of seeds, consists of 75–80% polyunsaturated fatty acids (PUFAs), of which 53–60% is linoleic acid (C18:2, n-6), 15–25% α-linolenic acid (C18:3, n-3) and 3–6% γ-linolenic acid (C18:3, n-6). Oleic acid (C18:1, n-9) contains hemp oil 8–15%. Hemp seed oil is also a rich source of tocopherols, which contains 1500 mg per kilogram [8,9]. The two major proteins present (edestin and albumin) are easily digestible and contain all essential amino acids [8,10]. In regards to protein quality, lysine is the first limiting amino acid in cannabis protein for several animals [11]. 

In addition to these essential nutrients, cannabis contains compounds such as plant sterols and phytocannabinoids, including the most abundant delta-9-tetrahydrocannabinol (THC), which is a strong fat-soluble antioxidant, stimulating appetite [12,13,14]. Cannabinoids are substances found only in cannabis. Cannabis contains more than 60 (phyto) cannabinoids that have anti-inflammatory, analgesic effects [15,16,17], anti-ischemic [18], antipsychotic, anxiolytic (against anxiety) [19] and effects against epilepsy [20]. Antimicrobial, immunomodulatory, antioxidant and antihypertensive effects are also described [14]. 

The best known and most studied of the cannabinoids is psychoactive tetrahydrocannabinol (THC), whose metabolite with potential immunosuppressive and anti-inflammatory effects that retains its psychoactive effects is cannabinol (CBN). Another metabolite of THC is the so-called cannabidiol (CBD), which no longer has psychoactive effects [21]. Other cannabinoids studied include dronabinol (DBN), nabilone, CBN, CBD, and cannabichromen (CBC) and cannabigerol (CBG). Cannabis cultivated in temperate climates contains less psychoactive cannabinoid THC, it also contains less of the second most common CBD that has no psychoactive effects [21]. The nutrient composition, and hence the composition of the cannabis ingredients and by-products, can vary considerably.

The literature points to the existence of a lipid signaling (or transmission) system (i.e., the endocannabinoid system (ECS)), which seems to significantly affect the production of reactive oxygen species, inflammation and subsequent tissue damage, and affects the body’s metabolic functions [22,23]. This system affects the immunological system [24,25] and has enormous therapeutic potential in a wide range of cancer diseases [26,27,28], pain, neurodegenerative and cardiovascular diseases up to obesity, metabolic syndrome, diabetes (diabetes mellitus) and diabetic complications [22,29]. 

As already mentioned, the endocannabinoid system is a central signaling receptor system affecting several biological processes. This system consists of a group of molecules known as endocannabinoids and the cannabinoid receptors to which these molecules bind [30]. Endocannabinoids are endogenous bioactive fat transporters [22]. Anandamide is one of the major endogenous cannabinoids produced by the body to date, and 2-arachidonoyl-glycerol. In addition to endocannabinoids, there are also phytocannabinoids that occur almost exclusively in cannabis seedlings and that also bind to cannabinoid receptors in the body [22,30]. Research into the endocannabinoid system has led to the discovery of two types of cannabinoid receptors—the cannabinoid-1 receptor (CB1) and the cannabinoid-2 receptor (CB2) [22]. These receptors are found in various parts of the body, but mostly in the central nervous system (CB1) and in the immune system (CB2). Both the endocannabinoids of the body and the phytocannabinoids contained in cannabis seeds fit into these cannabinoid receptors as locks. The binding of endocannabinoids or phytocannabinoids to CB1 or CB2 receptors regulates and affects physiological processes in the body and explains the wide therapeutic use of cannabis. Further research has gradually discovered endocannabinoid receptors in other organ systems of the body. In addition to the central nervous system and the immune system, the endocannabinoid system has also been discovered in the cardiovascular, gastrointestinal, genital and urinary systems [30].

Thus, the diverse physiological effects of cannabinoids are due to the existence of specific receptors distributed in some organs and systems of the body [21]. The ECS appears to play an important role in the development and control of type I diabetes. The exact mechanism of this system is still not fully discovered [22], although a hypothesis states that exogenous cannabinoids and the endo-cannabinoid system increase feed intake and improve weight gain in animals by activating central cannabinoid receptors (CB1) [29]. In addition, ECS activation has been found in obese people [31], which has led to adipogenesis, lipogenesis, hepatic steatosis and increased insulin resistance [32]. However, a mouse model has shown that a CB1 receptor antagonist is able to effectively reduce weight and thereby reduce possible metabolic risk factors [33].

## 2. The Seed Cakes as a Feed 

### 2.1. Chemical Composition of Seed Cakes

For the most part, the feed quality is determined by the quality of plants. The quality of the plant and its seeds (i.e., expellers) is determined by many factors, such as agricultural technology, soil quality, climate and weather or seed treatment and seed pressing. These factors affect the crop in terms of the composition of basic nutrients as well as the composition of active substances.

**Figure 1:**
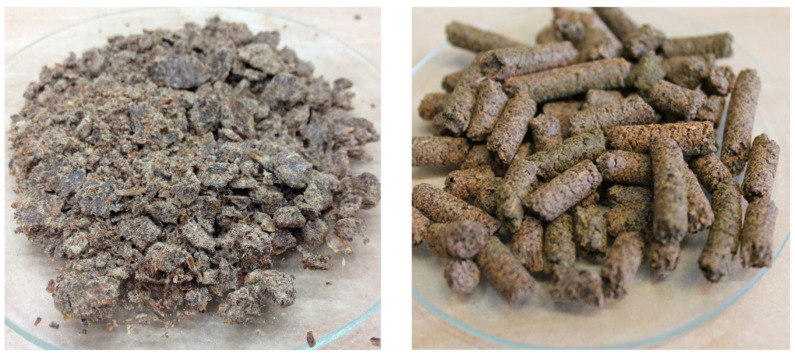
Seed cakes: (**a**) Milk thistle seed cakes are produced by seeds pressed through a hydraulic press; (**b**) hempseed cakes are formed by seeds extruded through a screw press.

Chemical composition of hempseed and milk thistle seed cakes used by us [34,35] are shown in Table 1. Generally, the seed cakes (i.e., expellers or pomace) are protein feed with residual 10% ether extract and relatively high amount of crude fiber. Figure 1 shows milk thistle seed cakes and hempseed cakes used in our experiments.

Many scientists have tried to demonstrate the effects of milk thistle in animal studies. It is believed that silymarin is mainly effective due to its anti-inflammatory and antioxidant effects, thereby stimulating hepatocyte regeneration [36]. For example, Kosina et al. [37] used milk thistle seed cakes in their experiment with rabbits containing 27% crude protein, 35% crude fiber, 10% fat, 6% starch and 4% of total flavonolignans (at 92% dry matter). This seed cakes contained more crude protein, more crude fiber and more total flavonolignans compared to ours. It was also reported [38] the following contents of the silymarin complex substances of the milk thistle seed cakes they used: 10.45 g/kg silychristin, 1.51 g/kg silydianin, 9.24 g/kg silybin A and 15.1 g/kg silybin B further 3.48 g/kg isosilybin A, 1.11 g/kg isosilybin B and 2.39 g/kg taxifolin. The contents of the substances of the silymarin complex found by the authors are much higher in comparison with ours, which could have been caused by different agricultural techniques, weather during the vegetation of the plant or by pressing the seeds themselves. In the study of Suchý et al. [39], it was stated that the milk thistle seed cakes contained 2.95% silymarin. For comparison, we present the results of Schiavone et al. [40],who used a silymarin extract containing 4.62% taxifoline, 28.21% silychristin + silydianin, 45.47% silybin isomers and 21.7% isosilybin isomers in the experiment.

Similarly, to milk thistle in animal nutrition, more scientists are studying cannabis in their work. Hemp seeds alone or in combination with hemp oil and by-products, such as seed cakes, have been included into animal diets [41,42,43,44]. In the publication of Halle and Schöne [44], hempseed cakes were used which, according to the analysis, contained (in 91% dry matter) 281 g/kg of nitrogenous substances, 110 g/kg of ether extract, 447 g/kg of neutral detergent fiber (NDF), 304 g/kg of acid-detergent fiber (ADF) and 117 g/kg of lignin. Serrapica et al. [45] determined a similar nutrient content of hempseed expellers. In contrast to the cannabinoid content, we have determined cannabidiol 170 mg/kg [34], and Halle and Schöne [44] state that the active substances content of THC and CBD was below the detection limit of 0.005%. The mineral nutrient content of hempseed cakes published by Halle and Schöne [44] is 28 g/kg calcium and 148 g/kg of phosphorus. 

### 2.2. Seed Cakes in Broiler Diets

In our experiments, from the fattening point of view, the live weight of chickens decreased where the content of seed cakes in the mixtures increased [34,46,47,48]. The trend of the lower live weight of chickens with an increasing proportion of expellers was also confirmed in the study of Suchý et al. [39], who found that the addition of 0.2% and 1% milk thistle expellers (2% crude fiber content in diets) led to a statistically insignificant reduction in live weight and feed conversion.

On the other side, Eriksson and Wall [43], in an experiment with Ross 308 in organic mode, found a significantly higher chickens live weight (1194 g) in the experimental group with 200 g/kg of hempseed cakes compared to the control group without hempseed expellers (1071 g), at 35 days of age. According to a study by di Marzo et al. [29], exogenous cannabinoids (ingested through a diet containing cannabis products) and the endocannabinoid system increase feed intake and improve weight gain in animals by activating central cannabinoid receptors (CB1 receptor). A summary of some experiments with Milk thistle seed cakes and Hempseed cakes in animal nutrition are given in Table 2 and Table 3, respectively.

It is important to realize the seed expellers contain approx. 35% of crude fiber. High levels of fiber in poultry nutrition cause digestive disorders and due to this worsened performance. For this reason, seed expellers must be limited in feed for chickens and hens. This means low content of active substances in the poultry diets. Many studies have confirmed the trend of a decreasing live weight in chickens with increasing crude fiber content in diets. The study of Halle and Schöne [44] showed worsened performance of animals with increasing level of crude fiber in the feed mixture. In many scientific publications with the results of experiments dealing with feeding predominantly expellers (or feeding diets containing a high crude fiber content), the authors do not report the crude fiber content in poultry diets [40,43,49,55,56], although the content of this nutrient had to be increased. Therefore, it is difficult to find a recommendation for the right amount of crude fiber in poultry diets, especially when the nutrient requirements standards [57,58] do not specify a crude fiber requirement in feed mixtures. Only few publications recommend the proportion of crude fiber in mixtures for poultry. The amount of 1–4% crude fiber for young poultry is recommended [59,60]. It was recommended up to 7% of crude fiber in the diet of laying hen [61]. The technological instructions for hybrids of hens from Lohmann Tierzucht [62] state the recommended crude fiber content in feed mixtures for laying hens to be 5–6%. On the contrary, the high content of predominantly soluble part of fiber in diet poultry can cause the deterioration of performance parameters, due to slower passage of feed through the digestive tract and reduced digestibility of nutrients [63].

### 2.3. Seed Cakes in Hens Diets

The experiment by Št’astník et al. [35] was performed on laying hens after 69 weeks of age to investigate the possible effect of milk thistle on their livers and performance—including egg quality parameters. Perspective results are found in the addition of 7% milk thistle seed cakes in the diet of laying hens. After the sixty-ninth week of age, the laying hens in the experimental group reached a higher number of eggs and produced more egg mass compared to the control group. In the evaluation of the egg quality parameters, higher Haugh units were found, a higher millimeter height of the egg, but a thinner shell in the laying hens receiving 7% of the seed cakes. When evaluating health indicators, higher antioxidant activity was found in the experimental group [35], which could be due to silymarin content. It has a polyphenolic structure which is the reason for its antioxidant properties. The hydroxyl groups of the silymarin complex have the potential to scavenge free radicals [64]. In older laying hens, summation of the liver tissue occurs during the laying cycle due to its own high performance. The use of milk thistle seed cakes (as a source of silymarin complex) for the experiment in laying hens was chosen for potential prevention liver damage and also due to the fact a higher content of crude fiber may be included in the feed mixture of laying hens [61]. In the experiment [35], 7% of milk thistle seed cakes were added to the mixture, which meant a content of 5.3% of crude fiber in the layers’ diet.

In our experiment [35], no differences in blood biochemical parameters were found with the inclusion of 7% milk thistle (i.e., no effect of milk thistle seed cakes on the monitored parameters of liver function or other selected parameters of laying hen metabolism was found). The same conclusion was reached by Blevins et al. [65], who added 1000 mg/kg of silymarin to the feed mixture of laying hens, but found no differences in the activity of the enzymes GGT and AST, but also in the activity of cytochrome (CYP) 450 34A. On the other hand, feeding different amounts of milk thistle flour in laying hens led to a significant reduction in the triglyceride levels in the blood of the animals compared to the control group [49]. The authors Hashemi Jabali et al. [49] further found that a dose of 60 g/kg of milk thistle flour reduced the concentration of blood cholesterol, and conversely increased the concentration of HDL (high density lipoproteins) cholesterol in the blood of animals (p > 0.05). The same conclusion was reached by Suchý et al. [39], who found a significantly lower concentration of blood cholesterol in fattened chickens at the forty-third day of age after the addition of 1% milk thistle to the feed mixture. The authors Gyenis et al. [66] published a range of plasma proteins in laying hens. This range for total protein content is between 30–50 g/L and 13–28 g/L for albumin, which almost corresponds to our results [35].

In a study of Neijat et al. [42], when feeding different proportions of hemp seeds (up to 30% in the feed mixture) and hemp oil (up to 9% in the feed mixture), the performance of laying hens (feed intake, weight gain, laying and egg weight) was not affected by dietary treatments. These results are consistent with previous studies on the use of cannabis products in laying hen nutrition [53,54]. These results may indicate the effect of the endocannabinoid system on animal performance. Unfortunately, study of Neijat et al. [42] do not state the cannabinoids content of the cannabis seeds used in trial. So, the question is how many cannabinoids substances the used seeds contained and how many of it the animals received in their diet. On the other hand, in the experiment where laying hens were fed (inter alia) hempseed expellers at 5%, 10% and 15%, a significantly lower performance (feed intake, egg production and consumption egg feed) was found at a level of 15% seed cakes in diet [44]. This reduction in performance parameters (or the fact that the expellers addition did not favorably affect the performance of the laying hens) could be due to the presence of low content or absence of cannabinoids in the seed cakes. The authors mention that the hempseed expellers used by them was almost free of cannabinoids (THC and CBD were found below the detection limit of 0.005%). The hempseed cakes used in our experiments contained 0.017% CBD. Further increase in the content of seed cakes in the diets led to a reduction in the proportion of yolk and an increase in the proportion of egg white [44], for which the mentioned proteins and the quality of their amino acids could be responsible.

## 3. The Milk Thistle and Hemp Seed Bioactive Substances Effects on Metabolism and Performance 

Many authors have tried to make milk thistle substances available to poultry without the need to feed expellers or other forms of plant by-products [40,50,67]. For example, Gawel et al. [67] fed chickens and turkeys with a commercial preparation containing 80% of silymarin at doses of 0.5 kg and 1 kg per ton of feed mixture. These authors found that the final live weight of the Cobb 500 chickens in the experimental groups was 4.8–6.6% higher than in the control group. Feed conversion ratio was balanced in all groups. The authors recorded the highest percentage of mortality (4.1%) in the chickens of the control group. Similarly, these authors Gawel et al. [67] found 1.9–3.8% higher live weight in BUT 9 hybrid turkeys fed mixtures containing the addition of silymarin compared to the control group. The turkeys were similarly affected, with a higher live weight of 2.5–3.0%. Schiavone et al. [40] found no differences in the live weight of chickens at the end of the experiment, but also in feed consumption and feed conversion when feeding a commercial milk thistle seed extract containing 40 and 80 ppm of silymarin. Another study by Zarei et al. [50] applied 1 mL of milk thistle extract in ovo in two dilutions (100 and 200 mg/L) and then also added it to the feed mixture at a dose of 100 mg/kg. They found that the level of 100 mg/L in ovo caused a significantly lower (*p* < 0.05) chicken’s final live weight (2125 g) at 42 days of age compared to the control group (2179 g). The feed mixture containing 100 mg/kg of extract caused significantly higher final live weight (2218 g) of chickens compared to the control group (2079 g). Silymarin also has a positive effect on feed intake, weight gain [51,68,69,70,71] and liver tissue morphology of chickens [69]. In addition, silymarin supplementation has a significant effect on meat quality and shelf life by increasing post-mortem oxidative stability [40].

The enzymes GGT and AST are considered physiological indicators of liver health. High gamma-glutamyl transferase enzyme activity in avian blood plasma is associated with hyperplasia and bile duct tumors [72]. It was claimed by Tedesco et al. [51] that silymarin content of 600 mg/kg in the diet can reduce the plasma GGT and AST activity in broiler chickens’ blood affected by aflatoxicosis. In addition, silymarin has been shown to reduce the activity of these enzymes in people with liver diseases [73] as well. In [39], significantly lowered activity of ALT and AST enzymes in the blood plasma of the experimental groups (0.2% and 1% of milk thistle seed cakes in the diets) was found, compared to the control group, on day 22 of the chicken fattening period. At day 43 of fattening, the lower activity of these enzymes in the experimental groups was not conclusive, but lower GGT enzyme activity and lower cholesterol levels were demonstrated in the group containing 1% seed cakes in the diet compared to the control group [39]. Similar results were achieved by Alassi and Allaw [74] with the addition 1 g/kg milk thistle seed powder in quail diet. A significantly lower level of cholesterol, glutathione (GSH), malondialdehyde (MDA), ALT and AST and a higher level glucose, total protein and globulin was found in the experimental group compared to the control group. Moreover, after the addition of 150 g/day of milk thistle seed cakes to dairy cattle [38], a higher AST enzyme activity was found, with other measured parameters GGT, LD, total protein, albumin, glucose, unesterified fatty acids, β-hydroxybutyrate, urea and bilirubin unchanged. In contrast to the above authors, our results in the inclusion of milk thistle seed cakes in the poultry diet did not affect the activity of liver-specific enzymes such as AST and GGT [35].

Neijat et al. [42] also found significantly lowered AST and GGT enzyme activities in laying hens with the addition of 10% and 20% of cannabis seeds into feed compared to the control group. The creatine kinase and electrolytes in the blood plasma were not affected by the experimental treatment. It might appear that the addition of cannabis seeds had a positive effect on the health of the liver tissue of the laying hens compared to the control group in the experiment [42]. The same results were achieved by Afzali et al. and Barani et al. [75,76]. On the other hand, these authors [75,76] found that groups of chickens fed a diet containing different proportions of hemp seeds and hemp seed extrudates increased the immunoglobulin G titer compared to the control group. Skřivan et al. [52] found that the dietary supplementation with 40 g/kg hempseed and 60 g/kg extruded flaxseed improved cockerel performance, meat and bone quality and deposition of alpha tocopherol in the liver. Another study, by Palade et al. [77], suggests the potential of hemp seed in sow nutrition. The diets containing 2% and 5% of hempseed improved the overall antioxidant status of the lactating sows and their progeny.

The studies with different hepatotoxic substances showed that silymarin has a multiple action as a hepatoprotective agent. Its antioxidant properties and cell regenerating functions, because of increased protein synthesis, are considered as the most important [2,78]. The bioactive complex (silymarin) can enter into the nucleus and act on RNA polymerase I enzymes and rRNA transcription, leading to increased ribosomal formation. This in turn accelerates the synthesis of protein and DNA [79] which increases the biosynthetic apparatus in the cytoplasm, thus leading to an increase in the synthesis rate of both structural and functional proteins. This stimulation may allow cells to counteract the loss of transporters and enzymes occurring under many pathological conditions. This action has important therapeutic implications in the repair of damaged hepatocytes and restoring of normal liver functions [73].

In addition, according to several studies silymarin may protect cells from oxidative (e.g., thermal) stress. The significant upregulation of oxidative stress biomarkers including MDA, TNF-like, IFN-γ and IL-1β genes was observed upon heat stress in chicken hepatocytes. Furthermore, antioxidant enzyme (superoxide dismutase, catalase, glutathione reductase) activities decreased. The silymarin (259 µM) was able to normalize the expression of all these biomarkers in heat-induced chicken hepatocytes [80]. From this, silymarin (silibinin) may upregulate and improve antioxidant defenses. In another in vitro system based on heat-induced chicken hepatocytes, silymarin (259 µM) affected heat shocks proteins (Hsp70) expression significantly, preventing its alleviation by heat stress [80]. Thus, there are many possible mechanisms by which silymarin may improve the body antioxidant defense mechanisms [81].

## 4. Conclusions

In the light of the above findings, it seems more appropriate to apply the extract of the bioactive substances complex to the livestock diets than addition of expellers or other forms of plants processing. The seed expellers etc. mostly worsened the chicken’s performance parameters with higher doses in diets. Therefore, it is better to include smaller proportions of these by-products in the diets of non-ruminant animals. However, most of the works using the extracts had positive results on animal performance. Although, when applying the extract to the diets, it is necessary to consider the higher costs to feed production.

## Figures and Tables

**Table 1:** Seed cakes chemical composition (dry matter basis).

Nutrient	Milk Thistle Seed Cakes	Hempseed Cakes
Gross energy (MJ/kg)	20.30 [35]	20.40 *
Crude protein (g/kg)	217.00 [35]	298.04 [34]
Ether extract (g/kg)	100.70 [35]	96.94 [34]
Crude ash (g/kg)	68.00 [35]	72.46 [34]
Crude fiber (g/kg)	292.40 [35]	325.53 [34]
ADF (g/kg)	413.80 [35]	420.64 *
NDF (g/kg)	455.40 [35]	478.11 *
β-carotene (mg/kg)	6.47 *	18.72 [34]
Cyanidine-3-glucoside (mg/kg)	129.83 *	46,62 [34]
Cannabidiol (mg/kg)	-	170 [34]
Tetrahydrocannabinol	-	non-detectable
Cannabinol	-	non-detectable
Taxifolin (mg/kg)	580 [35]	-
Silychristin (mg/kg)	3638 [35]	-
Silydianin (mg/kg)	2520 [35]	-
Silybin B (mg/kg)	6673 [35]	-
Silybin A (mg/kg)	1473 [35]	-
Isosilybin (mg/kg)	565 [35]	-

* results from our analysis (unpublished data); ADF—acid-detergent fiber; NDF—neutral detergent fiber

**Table 2:** The milk thistle seed cakes in poultry diets.

Animals		Exp. Duration	Type and Dose of Additive	Active Substances Concentration	Main Effect	Ref.
Species	n					
Hens, Bovans Brown	30	11 weeks (77 days)	Milk thistle seed cakes at 7%	Flavonolignans 37.3 mg/kg	higher number of eggs, more egg mass, higher antioxidant activity in the experimental group.	[35]
Rabbits, HYLA	360	42 days	*Silybum marianum* fruit 0.2% or 1%	Flavonolignans 4%	Mild effect on performance parameters.	[37]
Holstein cows	3	13 days	Milk thistle fruit expeller at 150 g/day/cow	4.10 ± 0.10 mass percentage of the silymarin complex	Animals receiving the milk thistle expeller had a higher content of plasma conjugated silybin.	[38]
Broiler chickens, ROSS 308	180	22, 43 and 52 days	*Silybum marianum* seed cakes 0.2% or 1%	2.95% of silymarin	Lower cholesterol, AST, ALT in exp. Groups.	[39]
Broiler chickens, ROSS 508	180	60 days	40 ppm and 80 ppm of a silymarin	Taxifolin—4.65%; Silychristin + Silydianin—28.21%; Silybin isomers—45.47%; Isosilybin isomers—21.7%	Silymarin at the tested doses did not affect growth performances but slightly affected slaughtering yields negatively.	[40]
Broiler chickens, ROSS 308	150	25 days	Milk thistle seed cakes at 5% and 15%	Flavonolignans 37.3 mg/kg	The milk thistle seed cakes do not worsen the sensory characteristic of breast or thigh meat of broilers and reflects optimal sensory quality traits.	[46]
Leghorn hens, Hy-Line W-36	200	70 days	Milk thistle meal 15%, 30%, 60%	470.64 mg gallic acid equivalent/g of the sample	Milk thistle meal has antioxidant effect, beneficial effects on ileal pathogenic bacteria, intestinal histological alterations and production and reduction of serum MDA	[49]
Broiler chickens, ROSS 308	360 eggs and 240 chickens	1–21 days and 22–42 days	100 mg/L and 200 mg/L in ovo and (or) 100 mg/kg of *Silybum marianum* extract in diet	Silymarin (≥80%) and Silybin isomers (≥30%)	Dietary feeding of the extract to broiler chickens increased immunity response under elevated temperatures, but in ovo feeding of the extract had no impact on immunity.	[50]
Broiler chickens	21	35 days	Aflatoxin B1 at 0.8 mg/kg of feed + silymarin phytosome at 600 mg/kg	Silymarin phytosome	Silymarin might be used in chickens to prevent the effects of aflatoxin B1 in contaminated feed.	[51]

Exp.—experiment; Ref.—references.

**Table 3:** The hempseed cakes in poultry diets.

Animals		Exp. Duration	Type and Dose of Additive	Active Substances Concentration	Main Effect	Ref.
Species	n					
Broiler chickens, ROSS 308	150	25 days	Hempseed cakes 5% and 15%	170 mg/kg of CBD	Hempseed cakes affect the colour and odor of broiler chicken’s meat, which is positive for the consumers.	[34]
Lohmann LSL-Classic	48	12 weeks (84 days)	Hempseed 10%, 20% or 30%; hempseed oil 4.5% or 9%	Only nutrient composition	Hempseed and hempseed oil are tolerated by hens and may suggest protective effect in liver damage.	[42]
Broiler chickens, ROSS 308	1200	70 days	Hempseed cake 100 and 200 g/kg	Average level of THC (1–1.5 g/kg dry weight)	It was not affected total performance parameters, mortality or microbiological measures.	[43]
Laying hens	216	168 days	Hempseed cake 50, 100 or 150 g/kg	The THC and CBD content was below the detection limit of 0.005%.	Up to 10% of hempseed cake do not negatively influenced the laying hens performance and provide the possibility of the enrichment of yolk fat with n-3 type PUFA.	[44]
Broiler chickens, ROSS 308	540	35 days	Hempseed 30, 40 and 50 g/kg in diets	Only nutrient and fatty acids composition	Hempseed in diet increases tibia strength. The dietary supplementation with 40 g/kg hempseed and 60 g/kg extruded flaxseed improves bird’s performance, meat and bone quality and deposition of α-tocopherol in the liver.	[52]
Hens, DeKalb	102	4 weeks(28 days)	Hempseed meal 50, 100 and 200 g/kg	Only nutrient content	No differences were found for egg production, feed consumption, feed conversion ratio, body weight or egg quality.	[53]
Hens, Bovans White	48	12 weeks(84 days)	Hempseed oil 4%, 8% and 12%; Hempseed 10% and 20%	Only nutrient and fatty acids composition	Up to a maximum level of 20% hempseeds and 12% hempseed oil does not affect laying hens’ performance and leads to the enrichment of the n-3 fatty acid content of eggs.	[54]

Exp.—experiment; Ref.—references; PUFA—polyunsaturated fatty acids.
